# Opposing roles of LTB_4_ and PGE_2_ in regulating the inflammasome-dependent scorpion venom-induced mortality

**DOI:** 10.1038/ncomms10760

**Published:** 2016-02-23

**Authors:** Karina F. Zoccal, Carlos A. Sorgi, Juliana I. Hori, Francisco W. G. Paula-Silva, Eliane C. Arantes, Carlos H. Serezani, Dario S. Zamboni, Lúcia H. Faccioli

**Affiliations:** 1 Departamento de Análises Clínicas, Toxicológicas e Bromatológicas, Universidade de São Paulo (FCFRP/USP), Ribeirao Preto, Sao Paulo 14040-903, Brazil; 2 Departamento de Biologia Celular, Molecular e Bioagentes Patogênicos, Universidade de São Paulo (FMRP/USP), Ribeirao Preto, Sao Paulo 14049-900, Brazil; 3 Departamento de Física e Química, Universidade de São Paulo (FCFRP/USP), Ribeirao Preto, Sao Paulo 14040-903, Brazil; 4 Department of Microbiology and Immunology, Indiana University School of Medicine, Indianapolis, Indiana 46202, USA

## Abstract

*Tityus serrulatus* sting causes thousands of deaths annually worldwide. *T. serrulatus*-envenomed victims exhibit local or systemic reaction that culminates in pulmonary oedema, potentially leading to death. However, the molecular mechanisms underlying *T. serrulatus* venom (TsV) activity remain unknown. Here we show that TsV triggers NLRP3 inflammasome activation via K^+^ efflux. Mechanistically, TsV triggers lung-resident cells to release PGE_2_, which induces IL-1β production via E prostanoid receptor 2/4-cAMP-PKA-NFκB-dependent mechanisms. IL-1β/IL-1R actions account for oedema and neutrophil recruitment to the lungs, leading to TsV-induced mortality. Inflammasome activation triggers LTB_4_ production and further PGE_2_ via IL-1β/IL-1R signalling. Activation of LTB_4_-BLT1/2 pathway decreases cAMP generation, controlling TsV-induced inflammation. Exogenous administration confirms LTB_4_ anti-inflammatory activity and abrogates TsV-induced mortality. These results suggest that the balance between LTB_4_ and PGE_2_ determines the amount of IL-1β inflammasome-dependent release and the outcome of envenomation. We suggest COX1/2 inhibition as an effective therapeutic intervention for scorpion envenomation.

## Abstract (web summary)

Scorpion venom causes thousands of deaths worldwide. Here the authors show that the envenomation acts via prostaglandin E2 leading to inflammasome activation and lung pathology, counterbalanced by inflammation-limiting LTB4, and that COX inhibitors or exogenous LTB4 rescue the venom-induced mortality in mice.

## Graphical abstract

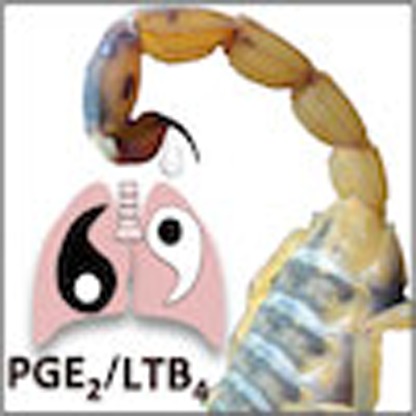

Incidences of scorpion stings have surpassed 1.2 million a year and are a serious health problem worldwide, resulting in severe wounds and more than 3,000 deaths annually^1^. Clinical manifestations of severe scorpion envenomation are local pain and multi-organ failure, including cardiogenic shock and pulmonary oedema^1^,^2^,^3^,^4^,^5^. Scorpion antiserum is the only available treatment, but it is not always effective and might induce adverse effects^1^,^4^. To develop a specific and effective treatment, it is necessary to understand the pathogenesis of the reaction induced by a scorpion sting.

Human envenomation by *Tityus serrulatus* venom (TsV) induces a massive and fast release of cytokines such as IL-1β, IL-6, IL-8, IL-10 and TNF-α factor^6^. Animal models of envenomation have been instrumental for the characterization of the inflammatory response and cell activation induced by TsV (refs 5, 7, 8). TsV or venom-associated molecular patterns (VAMPs) act in a way analogous to pathogen-associated molecular patterns (PAMPs) or damage-associated molecular patterns^9^,^10^ and are also recognized by pattern recognition receptors (PRRs) in leukocytes^11^. The recognition of TsV by PRRS induces NFκB and AP-1 or PPAR-γ activation, leading to the production of proinflammatory cytokines and eicosanoids^11^,^12^.

Inflammasome activation induces the production of lipid mediators^13^ and contributes to IL-1β secretion induced by bee venom^14^,^15^. Lipid mediators such as PGs and LTs are arachidonate (AA)-derived eicosanoids produced by cyclooxygenases 1/2 (COX1/2) and 5-lipoxygenase (5-LO), respectively, and are produced in abundance by resident and inflammatory cells^16^. PGE_2_ has important functions in innate and adaptive immune responses through the binding to one of its four different G-protein-coupled E-prostanoid (EP) receptors, known as EP-1-4 (ref. 17). The EP2 and EP4 are Gαs-coupled receptors that activate adenylate cyclase and cAMP production, while EP1 receptor increases intracellular calcium, and EP3 is a Gαi-coupled receptor that decreases cAMP formation^18^,^19^. PGE_2_ is a potent vasodilator and vascular permeability factor^20^,^21^ and induces gene expression of the inflammatory and edematogenic cytokine IL-1β via cAMP-PKA signalling pathway^22^. On the other hand, LTB_4_ does not induce oedema formation^23^; however, via GPCRs, BLT1 (high-affinity receptor) rather than BLT2 (low-affinity receptor)^24^ induces recruitment and activation of leukocytes^25^ and increases phagocytosis and killing of microorganisms by macrophages^26^. Interestingly, low concentrations of LTB_4_, but not LTD_4_, reduce intracellular cAMP production in macrophages^26^,^27^.

We found that intraperitoneal inoculation of mice with TsV induces the activation of the NLRP3 inflammasome, resulting in the production of IL-1β, LTB_4_ and PGE_2_, lung oedema and neutrophil recruitment and animal mortality. In summary, we identified that, in scorpion envenomation, a complex and intricate cAMP-mediated mechanism is responsible for lung oedema and mortality and unveiled a heretofore unknown cross-talk between IL-1β and eicosanoids, where the balance between 5-LO and COX1/2 products appears to determine the severity of envenomation.

## Results

### TsV induces the activation of the inflammasome

**Figure 1:**
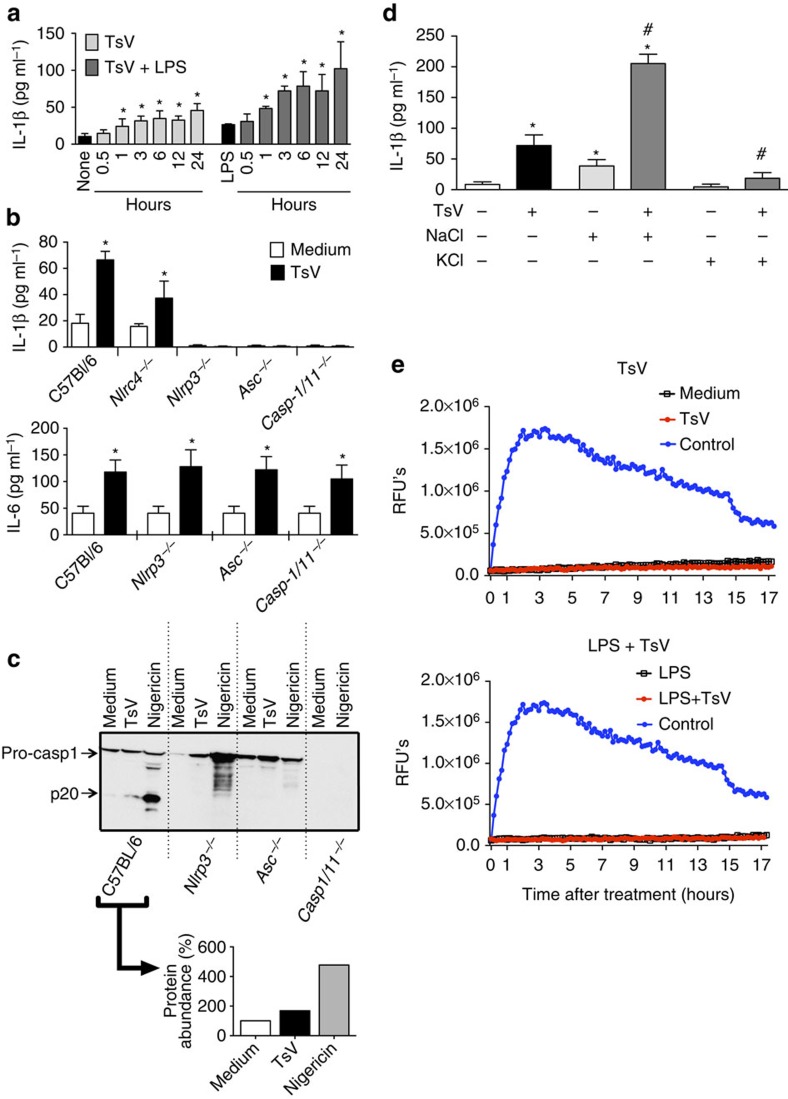
*T. serrulatus* venom (TsV) induces the activation of the NLRP3 inflammasome in macrophages. BMDMs from C57Bl/6 mice were pre-treated or not (**a**) with LPS (1,000 ng ml^−1^) for 4 h and then stimulated with TsV (50 μg ml^−1^) for 0.5, 1, 3, 6, 12 and 24 h to induce IL-1β release, which was measured by ELISA. (*n*=4) (**b**) IL-1β and IL-6 production measured by ELISA in the culture supernatant of BMDMs from C57Bl/6 WT, *Nlrc4*^*−/−*^, *Nlrp3*^*−/−*^, *Asc*^*−/−*^ and *Casp1/11*^*−/−*^ mice; the cells were incubated only with TsV (50 μg ml^−1^) for 24 h. (*n*=4) (**c**) C57Bl/6 WT, *Nlrp3*^*−/−*^, *Asc*^*−/−*^ and *Casp1/11*^*−/−*^ BMDMs were pre-treated for 4 h with LPS (1,000 ng ml^−1^) and later stimulated with TsV (50 μg ml^−1^) for 24 h or nigericin as a positive control (20 μM) for 1 h. Immunoblotting for caspase 1 (p20) in the culture supernatant was performed and the percentage of p20 abundance was calculated by densitometric analysis and is shown next to the panels. The data are representative of two independent experiments. (**d**) BMDMs from C57BL/6 mice were stimulated with TsV (50 μg ml^−1^) plus NaCl (50 mM) or KCl (50 mM) for 24 h and IL-1β was detected in the cell supernatant by ELISA. All data are representative of three independent experiments (*n*=3). (**e**) BMDMs from C57Bl/6 mice were pre-treated or not with LPS (1 μg ml^−1^) for 4 h and then stimulated with TsV (50 μg ml^−1^) or Triton-X (9%), which was used as the positive control for pore formation. Fluorometric plots show propidium iodide uptake (RFUs) over time to demonstrate the kinetics of pore formation in cell membrane. The data are representative of two independent experiments, performed in triplicate. Error bars (**a**,**b**,**d**), s.d. *P<0.05 (one-way ANOVA with Bonferroni's post-test compared with non-TsV-stimulated), ^#^*P*<0.05 (one-way ANOVA with Bonferroni's post-test compared with TsV-stimulated). Images have been cropped for presentation. Full size images are presented in Supplementary Fig. 6. RFU, relative fluorescence units.

Although bee venom activates the inflammasome to produce mature IL-1β (refs 14, 15), whether scorpion venom activates inflammasome remains unknown. We initially tested whether TsV induces inflammasome activation and IL-1β release. Bone marrow-derived macrophages (BMDMs) from C57BL/6 wild-type (WT) mice were either pre-treated or not with lipopolysaccharide (LPS) and incubated with a TsV concentration capable of activating macrophages *in vitro*^11^,^12^. TsV alone induces the significant production of IL-1β as early as 1 h after stimulation, demonstrating that the venom, *per se*, delivery signals are necessary for the activation of the inflammasome in BMDMs (Fig. 1a). Next, we determined which component of the NLRP3 inflammasome accounts for caspase-1/11 activation in response to TsV. Experiments with BMDMs deficient in various inflammasome constituents demonstrated that *Nlrp3*^*−/−*^, *Asc*^*−/−*^ and *Casp1/11*^*−/−*^ cells did not produce IL-1β (Fig. 1b) and did not activate caspase-1 in response to TsV (Fig. 1c). During the TsV challenge, macrophages from *Nlrc4*^*−/−*^ mice produced higher amounts of IL-1β than did macrophages from *Nlrp3*^*−/−*^ mice and lower amounts than those produced by macrophages from WT mice. These data suggest that NLRP3 and the NLRC4 inflammasome, to some extent, contribute to venom-induced IL-1β production (Fig. 1b). Western blotting analysis of the p20 subunit of caspase-1 (ref. 28) confirmed that TsV activated the NLRP3 inflammasome (Fig. 1c) as did nigericin, the positive control^28^. As a control, we determined the production of IL-6, an inflammasome-unrelated cytokine. As expected, BMDMs produced IL-6 in response to TsV regardless to the inflammasome components NLRP3, ASC and caspase-1/11 (Fig. 1b). To assess the mechanisms by which inflammasome triggers NLRP3 activation, we investigated the induction of pores in the macrophage membranes and the efflux of potassium, an essential process to enable the activation of the NLRP3 inflammasome^29^. We found that elevated concentrations of extracellular K^+^ impaired the inflammasome activation in response to TsV (Fig. 1d), suggesting that K^+^ efflux is involved in the activation of the inflammasome mediated by TsV. Notably, a higher concentration of NaCl potentiates TsV-induced IL-1β release (Fig. 1d), suggesting that the venom activates the inflammasome by a mechanism that is distinct from that induced by hyperosmotic stress, as reported previously^30^. To investigate whether TsV directly triggers cell death, we performed a pore-forming assay using WT-BMDMs treated with TsV in presence or absence of LPS. We verified that, even at 17 h after treatment, the cells did not present significant membrane damage, while cells treated with Triton-X, the positive control, did (Fig. 1e), confirming that TsV-induced cell activation without significant cell death.

### TsV induces lung inflammation and mortality via IL-1R/IL-1β

**Figure 2:**
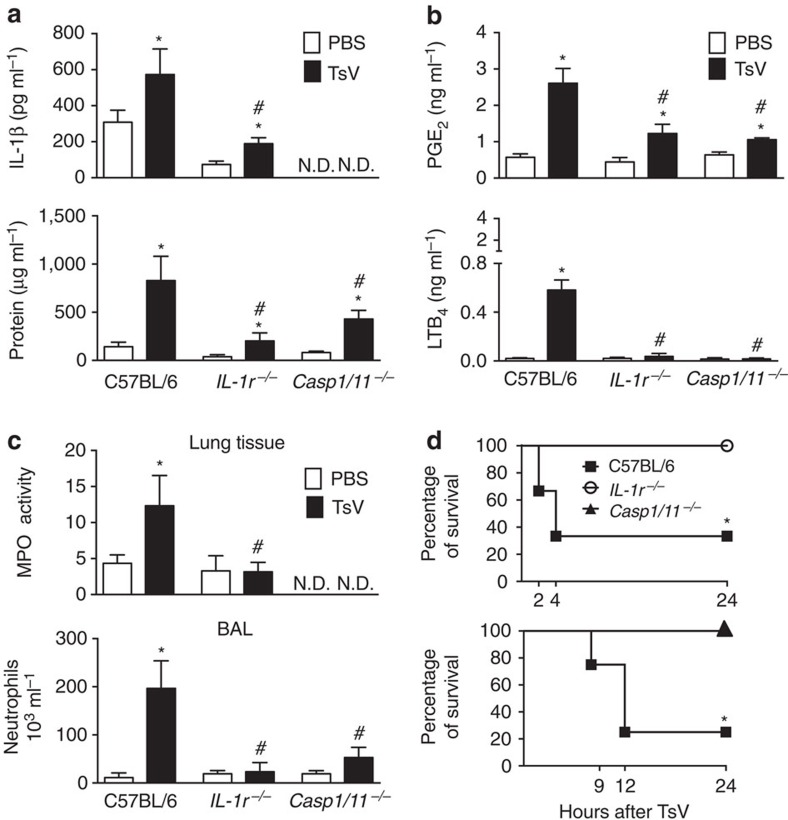
Lung inflammation and mortality induced by *T. serrulatus* venom (TsV) depend on IL-1R signalling and caspase-1/11 activation. (**a**–**c**) C57BL/6 WT, *IL-1r*^*−/−*^ or *Casp1/11*^*−/−*^ mice were inoculated intraperitoneally with a sublethal dose of TsV (120 μg kg^−1^) or PBS and the lungs were excised 4 h later for analysis of (**a**) IL-1β production and total protein, (**b**) PGE_2_ and LTB_4_ concentrations, (**c**) MPO activity and in a separate group of mice, BAL was performed to count neutrophils within the same period. (**d**) C57BL/6 WT, *IL-1r*^*−/−*^ or *Casp1/11*^*−/−*^ mice were inoculated intraperitoneally with a lethal dose of TsV (180 μg kg^−1^) for assessment of survival; mice were monitored for 24 h. (**a**–**c**) Experiments with a sublethal dose of TsV were performed once with six mice and error bars denote s.d. (**d**) Data on the lethal dose of TsV are shown as a percentage of surviving animals (*n*=6). *PBS versus TsV; ^#^ TsV in WT mice versus TsV in *IL-1r*^*−/−*^ mice or in *Casp1/11*^*−/−*^ mice. These differences were considered significant with *P*<0.05 according to one-way ANOVA with Bonferroni's post-test (**a**–**c**) or the log-rank test (**d**).

Because TsV activates the inflammasome *in vitro* and since in humans and animals, scorpion envenomation induces IL-1β (refs 5, 6), lung oedema^1^,^2^,^3^ and mortality^1^,^2^,^3^,^4^,^5^, we performed an *in vivo* dose–response experiment to identify the inflammatory, lethal and excessive doses of TsV for C57BL/6 and 129sv mice, which are the WT control mice for the inflammasome, and for *Alox5*^*−/−*^ mice, respectively (Supplementary Fig. 1). We used a sublethal dose of TsV (120 μg kg^−1^ intraperitoneally) to study inflammasome activation, IL-1β release and IL-1R signalling-induced lung inflammation *in vivo*. We collected bronchoalveolar lavage fluid (BAL) or whole lungs 4 h after envenomation from C57BL/6 (WT), *IL-1r*^*−/−*^ and *Casp1/11*^*−/−*^ mice. TsV activated inflammasome in lungs, inducing a significant increase in IL-1β release and protein extravasation (Fig. 2a), PGE_2_ and LTB_4_ production (Fig. 2b), and neutrophil recruitment to lung tissue and bronchoalveolar space in C57BL/6 mice (Fig. 2c). To evaluate if TsV induces the expression of enzymes involved in eicosanoid metabolism, peritoneal macrophages from C57BL/6 mice were incubated *in vitro* with TsV for 2 and 6 h and *Ptgs2*, *Ptges2, Alox5* and *Alox5ap* mRNA expression was determined. Interestingly, we observed that the mRNA expression of enzymes involved in the PGE_2_ biosynthetic pathway were increased earlier than mRNA for enzymes involved in LTs production (Supplementary Fig. 2). Activation of the IL-1β–IL-1R axis during the TsV-induced mediator release and lung inflammation was confirmed in *IL-1r*^*−/−*^ mice. After TsV inoculation, we detected lower levels of IL-1β and protein extravasation in *IL-1r*^*−/−*^ mice (Fig. 2a). Moreover, PGE_2_ and LTB_4_ production and neutrophil accumulation in lungs were lower in the absence of IL-1R than in WT mice (Fig. 2b,c). These results indicated that IL-1β/IL-1R signalling is essential for TsV-induced lung inflammation. Next, we determined whether the absence of IL-1β/IL-1R signalling protects mice from a lethal dose of TsV (180 μg kg^−1^). We found that 100% of *IL-1r*^*−/−*^ mice survived the lethal dose, while only 35% of the C57BL/6 mice survived (Fig. 2d). Oedema and cell accumulation induced by the lethal dose of TsV were also assessed macroscopically using Evans blue leakage assay and microscopically by haematoxylin and eosin (H&E) staining of lung tissue. Whole-lung staining in C57BL/6 mice showed intense Evans blue colouring and cell infiltration (Supplementary Fig. 3a,c), whereas the lungs from *IL-1r*^*−/−*^ mice did not stain positive for Evans blue and showed milder oedema and weaker infiltration by leukocytes (Supplementary Fig. 3d,f). As expected, TsV intraperitoneal inoculation induced neutrophil recruitment to the peritoneal cavity and increased the number of circulating neutrophils. However, these were lower in *IL-1r*^*−/−*^ mice (Supplementary Fig. 3g,h). To confirm the role of caspase-1/11 in the TsV-induced IL-1β secretion *in vivo*, we performed experiments using the *Casp1/11*^*−/−*^ mice. We found that caspase-1/11-deficient animals showed a reduction in protein extravasation (Fig. 2a), in PGE_2_ and LTB_4_ release (Fig. 2b), and in neutrophil recruitment to the lung and BAL (Fig. 2c) 4 h after administration of 120 μg kg^−1^ TsV. The impact of caspase-1/11 deficiency on mortality among the mice after lethal dose of TsV (180 μg kg^−1^) was examined and, up to 24 h, 100% of *Casp1/11*^*−/−*^ survived, while only 25% of WT mice survived (Fig. 2d). These data confirmed that caspase-1/11 and IL-1β are essential participants in lung inflammation and mediated animal death. Because we observed that *IL-1r*^*−/−*^ and *Casp1/11*^*−/−*^ mice still produced significant amounts of PGE_2_ after TsV administration, we speculated that PGE_2_ might be necessary for the release and the amplification loop for IL-1β production.

### 5-LO metabolites control lung inflammation and mortality

**Figure 3:**
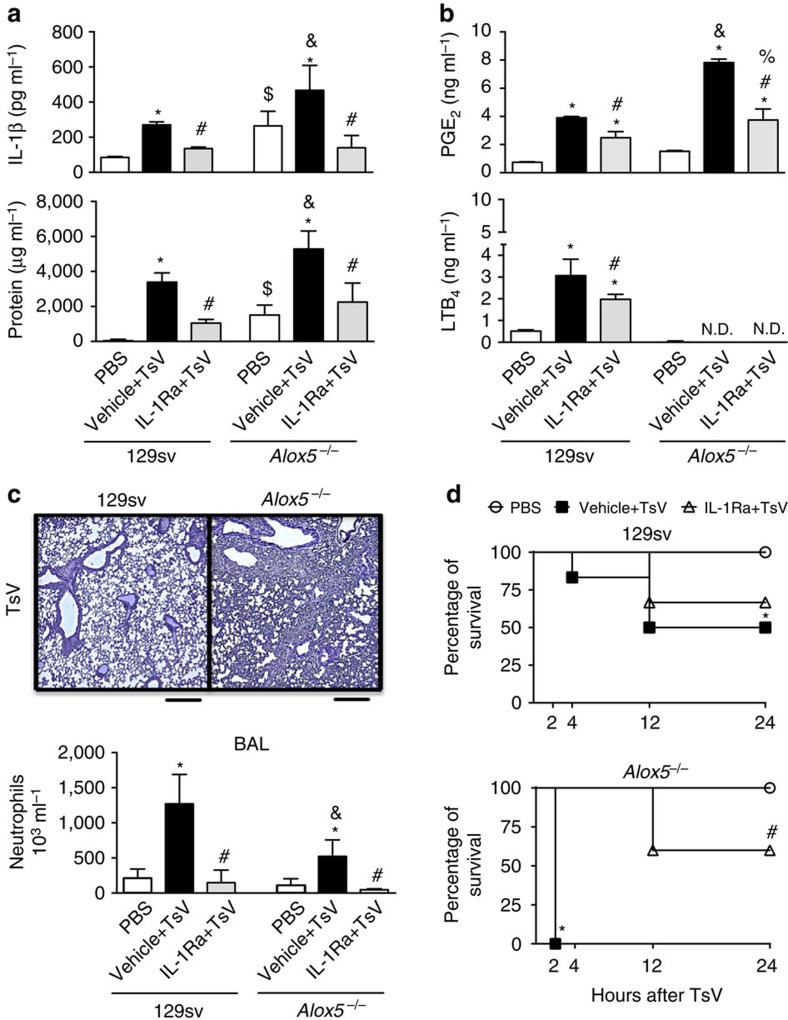
Downstream mediators of 5-LO protect mice from excessive lung inflammation and early mortality mediated by IL-1R signalling. (**a**–**c**) 129sv WT or *Alox5*^*−/−*^ mice were inoculated intraperitoneally with PBS or sublethal (120 μg kg^−1^) dose of TsV and lung tissues were recovered 4 h later for analysis of (**a**) IL-1β production and protein content; (**b**) PGE_2_ and LTB_4_ release; and (**c**) histological features of infiltration by inflammatory cells, as determined by staining with H&E, and the number of neutrophils was determined in BAL within the same period in another group of mice. (**d**) For survival analysis, 129sv WT or *Alox5*^*−/−*^ mice that received PBS or a lethal (180 μg kg^−1^) dose of TsV were monitored for 24 h. (**a**–**c**) A group (*n*=6) of 129sv WT or *Alox5*^*−/−*^ mice was treated with vehicle or IL-1Ra (10 mg kg^−1^ intraperitoneally) 1 h before and again 1 h after TsV injection and was subjected to the above analysis. (**a**–**c**) The experiment with a sublethal dose of TsV was performed once on six mice and the error bars denote s.d. (**d**) Data on the lethal dose of TsV are shown as a percentage of surviving animals among six mice. *PBS versus vehicle+TsV, ^&^vehicle+TsV in 129sv versus vehicle+TsV in *Alox5*^*−/−*^, ^#^129sv or *Alox5*^*−/−*^ inoculated with vehicle+TsV versus 129sv WT or *Alox5*^*−/−*^ inoculated with IL-1Ra+TsV; ^$^PBS in 129sv WT versus PBS in *Alox5*^*−/−*^. These differences were considered significant with *P*<0.05 according to one-way ANOVA with Bonferroni's post-test (**a**–**c**) or the log-rank test (**d**). The histological analyses were single-blinded. Scale bars, 100 μm.

Our data show that the earlier release of LTB_4_ was dependent on inflammasome, while PGE_2_ production was only partially dependent on the inflammasome (Fig. 2b). Therefore, we hypothesized that *Alox5*^*−/−*^ mice would develop a weaker lung inflammation with reduction in mortality after envenomation. Unexpectedly, when we performed a TsV dose–response curve (60, 120, 180 or 360 μg kg^−1^) and evaluated the survival up to 24 h, we observed that *Alox5*^*−/−*^ mice were more susceptible than 129sv and C57BL/6 mice (Supplementary Figs 1,4). We next investigated lung inflammation in *Alox5*^*−/−*^ and in WT 129sv mice, 4 h after injection of 120 μg kg^−1^ TsV. According to the increased susceptibility of these mice to TsV challenge, inflammation was stronger in the lung parenchyma of *Alox5*^*−/−*^ mice than that in the lung parenchyma of 129sv mice, as evidenced by an increase in IL-1β and protein concentration (Fig. 3a), PGE_2_ release (Fig. 3b) and leukocyte accumulation in the lung tissues (Fig. 3c). However, the number of neutrophils in BAL of *Alox5*^*−/−*^ mice was lower than that of BAL from 129sv WT mice (Fig. 3c). As expected, LTB_4_ amount in the lungs of 129sv mice increased after TsV injection (Fig. 3b). Because we observed a higher production of IL-1β in the lungs of *Alox5*^*−/−*^ mice (Fig. 3a), we next assessed the effects of exogenous IL-1 receptor antagonist (IL-1Ra) on TsV-induced lung inflammation and mortality. We found that, regardless of the mouse strain, IL-1Ra treatment decreased TsV-induced lung inflammation (Fig. 3a,b) and BAL neutrophil infiltration (Fig. 3c), confirming the crucial role of IL-1β for oedema and cell recruitment. Furthermore, treatment with exogenous IL-1Ra increased the rate of TsV-induced mortality among *Alox5*^*−/−*^ mice closer to that of 129sv IL-1Ra-treated mice. About 60% of the mice in both strains survived up to 24 h (Fig. 3d). To confirm that the high mortality of *Alox5*^*−/−*^ mice was not related to a nonspecific compensatory mechanism, we inoculated C57BL/6 mice with a lethal dose of TsV and either treated them or not with MK886, a leukotriene synthesis inhibitor. As expected, MK886 increased mortality, decreased LTB_4_ and IL-1β and increases PGE_2_ (Supplementary Fig. 5a–d). Taken together, these results indicate that mediators downstream of 5-LO activation protected the host from excessive inflammasome activation and consequently mortality after scorpion envenomation.

### COX1/2-EP2 axis promotes IL-1β-induced lung inflammation

**Figure 4:**
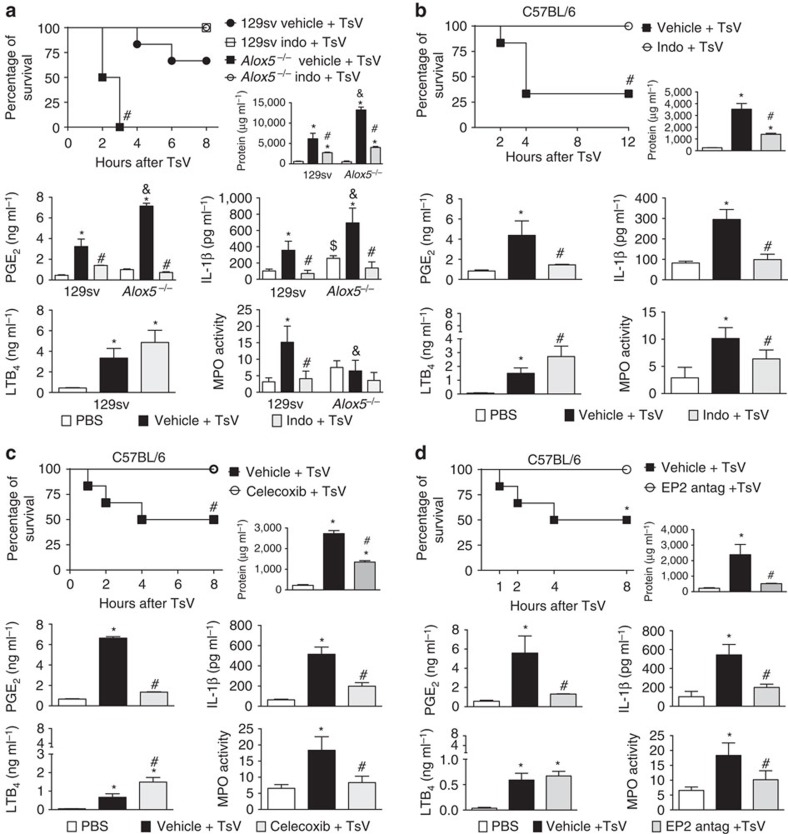
Inhibition of COX1/2, COX2 and PGE_2_-EP2 signalling decreases lung inflammation and abrogates mortality induced by a lethal dose of TsV. (**a**) 129sv WT mice and *Alox5*^*−/−*^ mice or (**b**) C57BL/6 mice were treated with indomethacin (Indo; 2 mg kg^−1^ intraperitoneally), 4 h and again 30 min before inoculation with a lethal (180 μg kg^−1^) dose of TsV. Additional administration of vehicle or indomethacin was performed 4 and 8 h later and the survival was monitored for 8–12 h. (**c**) C57BL/6 mice were treated with celecoxib (5 mg kg^−1^ intraperitoneally), or with (**d**) EP2 antagonist (AH6809) (5 mg kg^−1^ intraperitoneally), 24 h and again 1 h before inoculation of 180 μg kg^−1^ of TsV. In all experiments, TsV-inoculated mice treated with vehicle were used as controls. The lungs were excised immediately after an animal died or from mice that survived for 8–12 h; then protein content, PGE_2_ release, IL-1β production, LTB_4_ release and MPO activity were quantified in the lung parenchyma. The experiment was conducted once with six mice and the error bars denote s.d. *PBS versus vehicle+TsV; ^#^vehicle+TsV versus Treatments+TsV; ^$^PBS in 129sv WT mice versus PBS in *Alox5*^*−/−*^ mice; ^*&*^Vehicle+TsV in 129sv WT mice versus Vehicle+TsV in *Alox5*^*−/−*^ mice. These differences were considered significant with *P*<0.05, according to one-way ANOVA with Bonferroni's post-test (for soluble mediators and MPO) or the log-rank test (for survival).

As described above, 100% of *Alox5*^*−/*^ mice died after a lethal dose of TsV and produced 100% more PGE_2_ than 129sv mice did. Given that PGE_2_ induces IL-1β gene expression^22^,^31^ and oedema^20^,^21^, we next tested whether treatment of 129sv and *Alox5*^*−/−*^ mice with inhibitors that block both COX1 and COX2 (indomethacin) and COX2 alone (celecoxib) abrogates mortality induced by the lethal dose of TsV. Indomethacin abrogated TsV-induced mortality in both 129sv and *Alox5*^*−/−*^ mice and significantly inhibited protein extravasation, PGE_2_ and IL-1β production, and myeloperoxidase (MPO) expression in the lungs (Fig. 4a). Similar results were observed in C57BL/6 mice injected with a lethal dose of TsV and treated with indomethacin, celecoxib or an EP2 antagonist, AH6809. Although ∼50–70% of C57BL/6 mice died after administration of 180 μg kg^−1^ TsV, indomethacin, celecoxib and EP2 antagonist treatment decreased mortality, PGE_2_ and IL-1β production and neutrophil infiltration in the lungs (Fig. 4b–d). As expected, COX1/2 inhibition in C57BL/6 mice also resulted in increased LTB_4_ release (Fig. 4b,c). Inhibition of COX1 by SC-560 partially prevented TsV-induced mortality (Supplementary Fig. 5e). These results indicate that TsV-COX1/2-PGE_2_-EP2 signalling mediates excessive production of IL-1β, accounted for lung inflammation and is deleterious for the host.

### PGE_2_ increases IL-1β production via cAMP production

**Figure 5:**
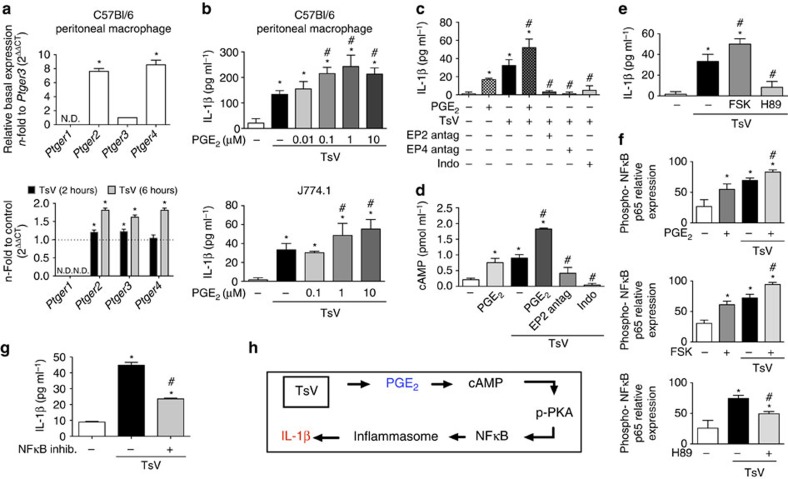
*T. serrulatus* venom (TsV)-induced macrophages IL-1β production is amplified by PGE_2_ via the cAMP pathway. (**a**) The relative mRNA expression levels for *Ptger1, Ptger2, Ptger3* and *Ptger4* are related to the expression level of *Ptger3* in resting macrophages of C57BL/6 mice. The fold increase in mRNA expression after TsV stimulation for 2 and 6 h was determined in comparison with that in non-stimulated cells. (**b**) Macrophages pre-treated with PGE_2_ (0.01–10 μM, 10 min), were stimulated with TsV and 24 h later supernatants were collected for IL-1β quantification. (**c**) J774.1 macrophages were pre-treated or not with EP2 (AH6809 1 μM), or EP4 (AH23848 1 μM) antagonists, or indomethacin (Indo 10 μM), for 30 min, followed or not by PGE_2_ (10 μM, 10 min), before TsV addition. Supernatants were collected 24 h later for IL-1β quantification. (**d**) cAMP in J774.1 macrophage pre-treatment or not with EP2 antagonist (AH6809 1 μM), or indomethacin (Indo 10 μM) for 30 min; followed or not by PGE_2_ (10 μM, 3 min), before TsV addition for 5 min. (**e**) IL-1β production by J774.1 macrophages pre-treated or not with PKA inhibitor (H89 25 μM, 2 h); or forskolin (FSK 25 μM, 10 min), before TsV addition for 24 h. (**f**) J774.1 macrophages were pre-treated or not with PGE_2_ (10 μM), or forskolin (FSK 25 μM) for 10 min; or with PKA-inhibitor (H89 25 μM, 2 h), and then stimulated or not with TsV. Cell lysates were obtained 2 h later for phospho-NFκB p65 (Ser536) and total NFκB p65 quantification. (**g**) J774.1 were pre-treated or not with NFκB inhibitor (20 nM, 30 min), before TsV and the supernatant was collected 24 h later for IL-1β quantification. The data are representative of two independent experiments performed in triplicate. Macrophages were stimulated with 50 μg ml^−1^ of TsV. (**h**) A schematic representation of the cell signalling pathway for IL-1β production induced by TsV is shown. (**a**–**g**) Significant differences *P*<0.05 are marked with symbols and the error bars denote s.d. *Medium only versus stimulus or treatments, and ^#^TsV versus treatments, according to one-way ANOVA with Bonferroni's post-test.

Previous studies showed that the PGE_2_-EP2 axis drives cAMP production^32^ and mediates IL-1β production^22^,^31^. First, we determined the relative basal expression of all EP receptors in resting macrophages and examined whether TsV upregulates the expression of EP1-EP4 receptors. We observed that non-stimulated macrophages exhibit a higher expression of EP2 and EP4, low expression of EP3 and no expression of EP1. Interesting, TsV increased the expression levels of *Ptger2-4* alone (Fig. 5a). Next, we investigated the effects of PGE_2_ on TsV-induced IL-1β and whether EP2/EP4 mediates cAMP increases, as previously described^33^. We observed that TsV-induced IL-1β expression in C57BL/6-peritoneal or immortalized macrophages (J774.1) was higher in PGE_2-_pre-treated cells and that indomethacin, the EP2 antagonist AH6809 and the EP4 antagonist AH23848 abrogated IL-1β production (Fig. 5b,c). To further evaluate the participation of PGE_2_-cAMP in TsV-induced IL-1β expression, we measured cAMP in the supernatant of J774.1 cells pre-treated as indicated. Interestingly, PGE_2_ alone increases IL-1β and cAMP (Fig. 5b,d). While forskolin, the adenylate cyclase agonist, potentiated TsV-induced IL-1β production, H89, the protein kinase A (PKA) inhibitor, reduced it (Fig. 5e). Moreover, PGE_2_ increased IL-1β production, while the EP2 antagonist AH6809 and indomethacin inhibited TsV-induced cAMP production (Fig. 5d). Next, we determined whether cAMP and its downstream effector PKA, leading to NFκB activation^34^,^35^, are involved in TsV-induced IL-1β. To do so, J774.1 cells were pre-treated with forskolin or with H89, and then challenge with TsV. We discovered that, in the presence of TsV, PGE_2,_ similar to forskolin, amplified the phosphorylation of NFκB p65, while the PKA inhibitor, H89, decreased it (Fig. 5f). The role of NFκB for TsV-induced IL-1β was confirmed by treatment with an NFκB inhibitor (Fig. 5g). Our results suggest that TsV via the PGE_2_-cAMP-PKA-NFκB pathway increases IL-1β production by inflammasome activation (Fig. 5h).

### LTB_4_ decreases cAMP generation and rescues mice from death

**Figure 6:**
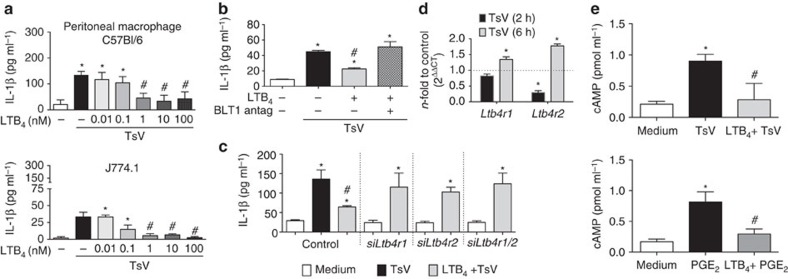
LTB_4_ decreases cAMP and IL-1β production induced by TsV. (**a**) Macrophages were pre-treated with LTB_4_ (0.01–100 nM, 10 min), before TsV (50 μg ml^−1^) addition for 24 h. Supernatants were collected for IL-1β quantification. (**b**) J774.1 macrophages were pre-treated or not with BLT1 antagonist (U-75302, 1 μM, 30 min) and with LTB_4_ (100 nM, 10 min) or not before TsV (50 μg ml^−1^) addition for 24 h. Supernatants were collected for IL-1β quantification. In another set of experiments, (**c**) J774.1 macrophages were transfected for 48 h to specifically silence *Ltb4r1* and *Ltbr4r2* gene expression. Cells were then pre-treated or not with LTB_4_ (100 nM, 10 min), before TsV (50 μg ml^−1^) addition for 6 h. In other experiment, (**d**) *Ltb4r1* and *Ltb4r2* mRNA expression in C57BL/6 peritoneal macrophages was determined following TsV stimulation (50 μg ml^−1^) at 2 and 6 h by qRT–PCR. (**e**) cAMP was determined following stimulation with TsV (50 μg ml^−1^, 5 min) or PGE_2_ (1 μM, 3 min); or LTB_4_ (10 nM, 1 min) followed by TsV or PGE_2_. (**a**–**e**) Data are representative of two independent experiments performed in quadruplicate. Significant differences *P*<0.05 are marked with symbols and the error bars denote s.d. *Medium only versus stimulus or treatments, and ^#^TsV versus treatments, according to one-way ANOVA with Bonferroni's post-test.

**Figure 7:**
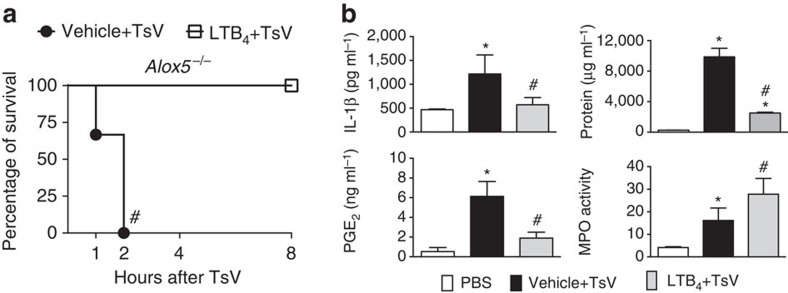
LTB_4_ rescues envenomed mice from mortality. (**a**) For assessment of survival and (**b**) inflammation in the lung, *Alox5*^*−/−*^ mice receiving a lethal dose of TsV (180 μg kg^−1^) were monitored for 8 h with or without exogenous LTB_4_ administration (50 ng per mice, intranasal). Lungs were then excised for analysis of total protein content, production of IL-1β, PGE_2_ release, and MPO activity. The experiment was conducted once with six mice and the error bars denote s.d. *PBS versus Vehicle+TsV; ^#^Vehicle+TsV versus LTB_4_+TsV. These differences were considered significant with *P*<0.05, according to one-way ANOVA with Bonferroni's post-test (for soluble mediators and MPO) or the log-rank test (for survival).

The abovementioned results indicate that PGE_2_ increases TsV-induced IL-1β by increasing cAMP production. We previously demonstrated that LTB_4_, but not LTD_4_, reduces cAMP generation in macrophages^27^. Thus, we hypothesized that LTB_4_ might play an anti-inflammatory function rather than being an inflammatory mediator during scorpion envenomation, by inhibiting NFκB activation. Our results show that LTB_4_ decreased IL-1β in both C57BL/6-peritoneal and immortalized (J774.1) macrophages (Fig. 6a) via BLT1/2 cell signalling, as evidenced by BLT1 antagonist (Fig. 6b) or receptor-gene silencing (Fig. 6c). Furthermore, TsV increases *Ltb4r1* and *Ltb4r2* mRNA expression in macrophages (Fig. 6d). As expected, LTB_4_ decreased cAMP-induced by TsV or by PGE_2_ (Fig. 6e). To corroborate the anti-inflammatory action of this lipid, lethally envenomed *Alox5*^*−/−*^ mice were treated with LTB_4_ as indicated. Exogenously administered LTB_4_ rescued *Alox5*^*−/−*^ mice from mortality (Fig. 7a) and decreased lung inflammation as noticed by the production of IL-1β and PGE_2_ and protein extravasation, besides increasing neutrophils recruitment to the lungs (Fig. 7b).

### COX1/2 inhibition rescues animals from lethal envenomation

**Figure 8:**
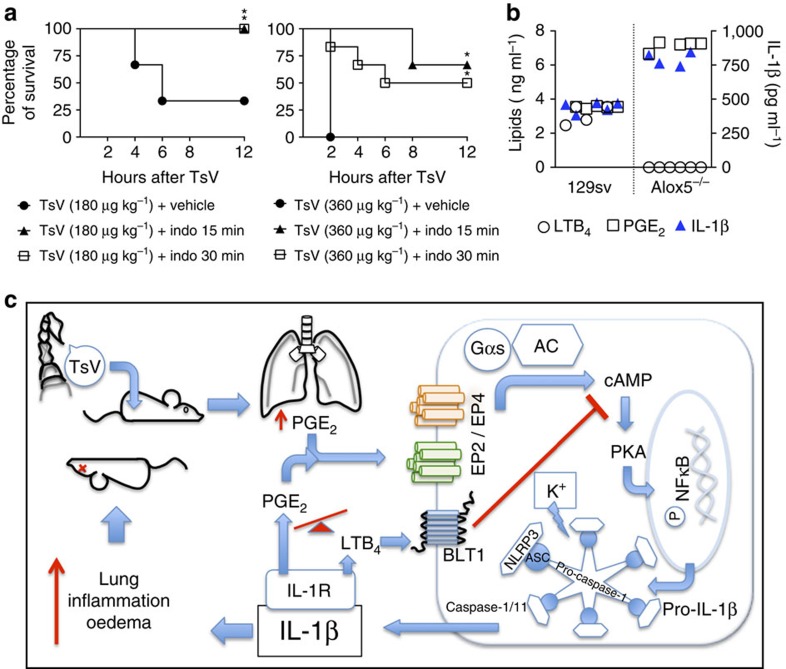
Treatment with an inhibitor of COX1/2 protects mice from scorpion envenomation. (**a**) C57BL/6 mice were inoculated with a lethal (180 μg kg^−1^) or excessive (superdose; 360 μg kg^−1^) dose of TsV and were treated with vehicle or indomethacin (Indo, 2 mg kg^−1^) 15 or 30 min after the venom injection, followed by an additional dose of the treatment 4 and 8 h later. Survival was monitored for 12 h. Significant differences (*P*<0.05) are marked with an asterisk. The experiment was performed once on six mice and the log-rank test was used to compare TsV+vehicle versus TsV+Indo. (**b**) Using the eicosanoid data obtained from the animals (129sv WT and *Alox5*^*−/−*^) inoculated with TsV at 180 μg kg^−1^ for 24 h, we demonstrated the correlation between the PGE_2_/LTB_4_ balance and the IL-1β production level, in each mouse. Data are representative of one experiment on six mice. (**c**) Mechanism scheme showing the production of eicosanoids after scorpion envenomation, inflammasome activation and IL-1β release, and indicating that the balance between metabolites of PGE_2_ and LTB_4_ determines the outcome of inflammasome-mediated envenomation, the severity of lung inflammation and the outcome of envenomation mortality.

Overproduction of PGE_2_-cAMP-IL-1β accounts for uncontrolled lung oedema and mortality in lethal envenomation. Thus, we next investigated whether COX1/2 inhibition can be used as a therapeutic treatment of scorpion stings. For this purpose, we injected C57BL/6 mice with a lethal dose (180 μg kg^−1^) or superdose (360 μg kg^−1^) of TsV and then therapeutically treated the animals with indomethacin as indicated. Under both regimens, indomethacin rescued the mice from the mortality induced by the lethal dose. Moreover, indomethacin strongly inhibited the envenomation by the superdose: 67% and 50% of the mice survived under the regimens ‘15' and ‘30 min,' respectively (Fig. 8a). As our data suggested that the balance between PGE_2_ and LTB_4_ regulates the amount of IL-1β produced and lung oedema (Supplementary Table 1), we next calculated the ratio of PGE_2_ to LTB_4_ in mice that received TsV at 180 μg kg^−1^ (Fig. 8b). Our data showed that the PGE_2_/LTB_4_ ratio affects IL-1β production *in vivo* and that when LTB_4_ is low, higher levels of PGE_2_ and IL-1β are produced and that a positive correlation exists between PGE_2_ and IL-1β. As illustrated in Fig. 8c, our data show for the first time that, during scorpion envenomation, an intricate and complex network controls lung oedema, cell recruitment and the stung animal's fate. Altogether, these results indicate that COX1/2 inhibition might be an effective treatment for patients stung by a scorpion in emergency rooms.

## Discussion

Bee venom activates the inflammasome^14^,^15^, resulting in eicosanoid production^13^,^14^. However, whether scorpion venom-induced pulmonary oedema and mortality in humans^1^,^2^,^3^,^4^,^5^ and animals^5^,^7^ are determined by inflammasome-dependent production of bioactive lipids remains unknown. We hypothesized that TsV activates the inflammasome and induces IL-1β, LTB_4_ and PGE_2_ production leading to lung oedema and mortality. In this study, macrophage-derived NLRP3 inflammasome was identified as a platform essential for a systemic response to TsV, resulting in PGE_2_, IL-1β and LTB_4_ production, lung inflammation and mortality.

Treatment with IL-1Ra or experiments with *Casp1/11*^*−/−*^ mice confirmed that the inflammasome is also activated by TsV *in vivo*, resulting in the production of IL-1β and PGE_2_, which are key determinants of the envenomation. The ability of IL-1Ra to control inflammation was reported previously^36^ and blocking IL-1β along with IL-1Ra has been proposed as an effective treatment of human inflammatory diseases^37^. Moreover, PGE_2_ upregulates IL-1β (refs 22, 31), and both of these mediators promote vascular permeability and oedema^20^,^21^,^38^, whereas IL-1β recruits neutrophils^39^. To the best of our knowledge, the present report is the first to demonstrate that IL-1Ra protects mice from envenomation, due to the diminished production of PGE_2_ and IL-1β and the consequent reduction of lung oedema.

The results demonstrating that the NLRP3 inflammasome is necessary for the LTB_4_ release led us to hypothesize that animals deficient in mediators downstream of 5-LO would be unaffected by scorpion envenomation, because it was previously described that LTs are necessary for the recruitment and activation of polymorphonuclear cells and for pulmonary oedema^40^,^41^. However, *Alox5*^*−/−*^ animals show overproduced PGE_2_ and IL-1β in the lungs leading to strong oedema and leukocyte infiltration in the tissue. Data from the literature demonstrated that PGE_2_ causes an array of beneficial and deleterious effects during inflammation^42^. Among these effects, PGE_2_ presents lung protective effects during chronic inflammation^43^,^44^. However, induction of oedema by PGE_2_ (refs 20, 21) (oedema and leukocytes recruitment by IL-1β (refs 38, 39)) is very well documented. The finding that *Alox5*^*−/−*^ mice are more sensitive to TsV than 129sv and C57BL/6 mice are underscores the protective role of LTB_4_ during scorpion envenomation. The recovery of envenomed *Alox5*^*−/−*^ mice by exogenously administered LTB_4_ confirmed its antiedematogenic role, although we cannot disregard a potential role of others eicosanoids such as lipoxins^45^ in antiedematogenic effects. In fact, we previously suggested that LTs could present antiedematogenic effects^46^. This was the first demonstration that LTB_4_ acts in an autocrine and paracrine manner as an antiedematogenic mediator by controlling PGE_2_ and IL-1β production, via cAMP-PKA-NFκB inhibition. Interestingly, our data also indicate that, in scorpion envenomation, IL-1β accounts for neutrophil recruitment to the lungs, but not to the bronchoalveolar space. In fact, decreased IL-1β concentration in *IL-1r*^*−/−*^*, Casp1/11*^*−/−*^ and IL-1Ra-, indomethacin-, celecoxib- and EP2-treated mice correlated with diminished MPO activity in the lung. Although our data indicate that LTB_4_ also regulates the intensity of neutrophil recruitment to the lung parenchyma by controlling IL-1β production, methodologically it is difficult to prove, since as observed in Fig. 7b and as previously described^25^, LTB_4_, *per se*, induces neutrophil recruitment. Altogether, our results show that, during scorpion envenomation, a cross-regulation mechanism exists between PGE_2_, IL-1β, and LTB_4_.

According to our data and to one report showing that a high dose of indomethacin decreases the severity of lung oedema induced in rats by one purified scorpion toxin (tityustoxin TsTX)^7^, we hypothesized that inhibition of COX1/2 or COX2 or blocking EP2/EP4-PGE_2_ interaction would result in the downregulation of PGE_2_ and IL-1β and would abrogate the lethality of TsV envenomation. These treatments increased LTB_4_ production and downregulated IL-1β and PGE_2_ in the lung and abrogated the mortality induced by a lethal dose of scorpion venom. Moreover, treatment with low dose of indomethacin rescued mice from the death induced by a lethal dose of TsV and significantly increased the survival rate after a high dose of TsV. In this context, we found that the ratio of 5-LO to COX products determined the amount of IL-1β released and the outcome of the envenomation. Furthermore, exacerbated lung inflammation and greater synthesis of IL-1β (ref. 47) and PGE_2_ (ref. 48) were previously observed in the presence of reduced level or in the absence of 5-LO products, but the effects of the augmented production of both mediators were not elucidated.

In summary, we can suggest that, in scorpion envenomation, PGE_2_ is produced in two waves. The first wave comes from resident macrophages and is inflammasome-independent, but involves the TLR4/MyD88-dependent activation of NFκB and TLR4-dependent activation of AP-1 (ref. 11). Then PGE_2_-EP2/EP4 actions activate cAMP-PKA-NFκB and triggers inflammasome activation and IL-1β release. The inflammasome-mediated IL-1β maturation signals via IL-1R in macrophages and induces the second and more prominent wave of PGE_2_ and discrete amounts of LTB_4_. Inflammasome-dependent PGE_2_ production in turn acts on EP2/EP4 receptors on macrophages (and perhaps neutrophils) and drives IL-1β production via the COX1/2-cAMP-PKA-NFκB axis. Meanwhile, TsV, sensed by TLR2, TLR4 and CD14 on macrophages induces neutrophil chemoattractants such as TNF-α, IL-6 and LTB_4_ (refs 11, 39). Therefore, LTB_4_, supplied by resident macrophages and newly recruited neutrophils, has autocrine and paracrine effects and, via BLT1/2, downregulates cAMP, restrains PGE_2_ and IL-1β production, consequently controlling lung oedema and possibly neutrophil recruitment. However, in contrast to our results, the regulation of NLRP3 inflammation by cAMP was previously demonstrated^49^, suggesting that more studies are needed. We propose that, in non-severe envenomation, the production of mediators is self-controlled and the patient presents discrete lung oedema, but survives. However, in severe envenomation, the production of PGE_2_ and IL-1β is highly induced and LTB_4_ delayed production is not sufficient to control the oedema and the patient succumbs.

Our findings may open new avenues of research and we speculate that patients with low levels of LTB_4_ or high levels of PGE_2_, which is the case for patients with immunodeficiencies^50^,^51^, are more likely to die after scorpion envenomation. Given the fact that many non-steroidal drugs are safe to use in humans, we anticipate that therapeutic strategies aiming to block PGE_2_ synthesis during envenomation would be effective, safe, and low-cost attractive therapies.

## Methods

### Animals

Female or male mice (6–8 weeks old) were used for *in vivo* and *in vitro* experiments. The mice were matched by sex and age in all procedures. Sample size was determined based on previous studies from our laboratory and literature and considering an alpha and beta errors of 0.05 and 0.20, respectively. *Nlrp3*^*−/*−^ (ref. 52), *Il-1r*^*−/−*^(ref. 30), *Nlrc4*^*−/−*^ (ref. 30), *Casp1/11*^*−/−*^ (ref. 53) and *Asc*^*−/−*^ (ref. 54) mice were backcrossed with C57BL/6 mice for at least nine generations and were obtained from the animal facilities of the Faculdade de Medicina de Ribeirão Preto (FMRP/USP). C57BL/6 mice also were obtained from the animal facilities of the Faculdade de Ciências Farmacêuticas de Ribeirão Preto, FCFRP/USP. 5-LO-deficient mice (*Alox5*^*−/−*^) (ref. 55) and 129sv WT strain were obtained from the Jackson Laboratory (Bar Harbor, ME, USA) and raised at FCFRP/USP. Maintenance of and experiments with the mice were in compliance with the institutional guidelines on ethics in animal experiments approved by the Animal Care Committee of the Prefeitura of the Campus of Ribeirão Preto (PCARP) at the University of São Paulo, Ribeirão Preto, Brazil (protocol number 14.1.272.53.7).

### *T. serrulatus* venom

The venom was extracted from several *T. serrulatus* scorpions by electric stimulation, dried at room temperature in a vacuum desiccator, and stored at −20 °C. Before the experiments, TsV was diluted in PBS and filtered through a 0.22-μm sterilizing membrane (Millipore, Tullagreen, CO, EUA). *Limulus* Amebocyte Lysate Test (LAL; QCL-1000, Bio Whittaker, Cambrex Company, Walkersville, MD, USA) was performed to detect LPS in the TsV samples, according to the manufacturer's instructions. No LPS was detected in any TsV samples.

### Activation of BMDMs and peritoneal macrophages by TsV

BMDMs were isolated from C57BL/6 (WT), *Nlrc4*^*−/−*^, *Nlrp3*^*−/−*^, *Asc*^*−/−*^ and *Casp*1/11^*−/−*,^ mice as described previously^56^. After differentiation, the culture medium was replaced with RPMI 1640 containing 10% (v/v) fetal bovine serum and 5% (v/v) of L929-cell conditioned medium. BMDMs were seeded (2 × 10^5^ per well in 1 ml) in a 24-well plate and either pre-treated or not-pre-treated with *Escherichia coli* LPS (1,000 ng ml^−1^; Sigma-Aldrich, St Louis, MO, USA) for 4 h and then incubated with TsV (50 μg ml^−1^) for 24 h at 37 °C in a humidified atmosphere containing 5% of CO_2_. Next, the supernatants were harvested for IL-1β and IL-6 quantification using an ELISA kit (R&D Systems, Minneapolis, MN, USA). In another set of experiments, resident peritoneal macrophages from naive C57BL/6 mice, harvested by peritoneal washes with RPMI-1640 and the J774.1 cell lineage of peritoneal macrophages, obtained from cell culture were plated at the density of 2 × 10^5^ cells per well in 200 μl of incomplete RPMI-1640 supplemented with antibiotics. The cells were then cultured at 37 °C (5% CO_2_) for 2 h. Next, the supernatants were removed and the cells were pre-treated or not with either LTB_4_ for 10 min (0.01, 0.1, 1, 10 or 100 nM; Cayman Chemical, Ann Arbor, MI, USA)^57^,^58^ or PGE_2_ (0.01, 0.1, 1 or 10 μM; Cayman Chemical)^59^ or forskolin^60^ (50 μM; Sigma-Aldrich) in 200 μl of incomplete RPMI. LTB_4_ and PGE_2_ from ethanol stock solution were diluted in the cell culture medium and the same concentration of ethanol (maximum 0.1%) was added to the medium only (control). Forskolin from DMSO stock solution was diluted in the cell culture medium and the same concentration of DMSO (maximum 0.1%) was added to medium only (control). Next, the cells were stimulated with TsV (50 μg ml^−1^) under the same experimental conditions, and after 24 h at 37 °C in a humidified atmosphere containing 5% of CO_2_, supernatants were collected for IL-1β quantification.

### Western blot analysis

BMDMs from C57BL/6 (WT), *Nlrp3*^*−/−*^, *Asc*^*−/−*^ and *Casp*1/11^*−/−*^ mice were seeded at 10^6^ per well, pre-treated with ultrapure LPS (1,000 ng ml^−1^; Sigma-Aldrich) for 4 h and then either incubated with TsV (50 μg ml^−1^) for 24 h or stimulated with 20 μM nigericin^28^ (Sigma-Aldrich) for 1 h at 37 °C (5% CO_2_). The supernatants were collected and suspended with the Laemmli buffer, boiled, resolved by SDS–polyacrylamide gel electrophoresis (15% gel) and transferred (Semidry Transfer Cell, Bio-Rad, Hercules, CA, USA) onto a 0.22-μm nitrocellulose membrane (GE Healthcare, Madison, WI, USA). The membranes were blocked with Tris-buffered saline containing 0.01% Tween 20 and 5% non-fat dry milk. The rat monoclonal antibody to caspase 1 (p20) clone 4B4 (Genentech) and specific horseradish peroxidase-conjugated antibodies (1:3,000; KPL, Gaithersburg, MD, USA) were diluted in blocking buffer for the incubation. The Enhanced Chemiluminescence Luminol Reagent (GE Healthcare) was used for antibody detection. Densitometric analyses were performed by using the Image J 1.34 s Software (National Institutes of Health, Bethesda, MD, USA). The quantitative densitometric data were expressed as a percentage of an increase relative to baseline control levels.

### Pore formation assay and inhibition of the K^+^ efflux

BMDMs (1 × 10^5^) were plated in 96-well tissue culture dishes for 16 h at 37 °C, 5% CO_2_. Next, cells were stimulated with LPS (1 μg ml^−1^) during 4 h and then treated with 50 μg ml^−1^ of TsV venom for 17 h. Pore-forming activity was measured by the quantification of propidium iodide uptake^61^. As a positive control, the cells were treated with Triton-X (9%). Throughout the treatment, plates were incubated at 37 °C in a SpectraMax fluorimeter plate reader and the propidium iodide fluorescence was measured every 10 min. For the inhibition of the K^+^ efflux, BMDMs from C57BL/6 mice were stimulated with TsV (50 μg ml^−1^) plus NaCl (50 mM) or KCl (50 mM) for 24 h (ref. 62). The supernatant was used to detect IL-1β by ELISA.

### *In vitro* pharmacological treatments

J774.1 macrophages were plated at the density of 2 × 10^5^ cells per well in 200 μl of serum-free RPMI supplemented with antibiotics. The cells were then cultured at 37 °C in 5% CO_2_ for 2 h. Next, the supernatants were removed, and the cells were treated or not with specific inhibitors/antagonists for 30 min: indomethacin (10 μM; Cayman Chemical); AH6809 (1 μM; Cayman Chemical); AH23848 (1 μM; Cayman Chemical); U-75302 (0.1 and 1 μM; Cayman Chemical); and NFκB Activation Inhibitor (20 nM; Calbiochem, Darmstadt, Germany). H89 dihydrochloride hydrate (25 μM; Sigma-Aldrich) was added for 2 h in the cell culture medium before stimulation. AH6809 and U-75302 from ethanol stock solutions were diluted in cell culture medium and the same concentration of ethanol (maximum 0.1%) was added to the medium only (control). The AH23848 and NFκB inhibitor from DMSO stock solutions were diluted in the cell culture medium and the same concentration of DMSO (maximum 0.1%) was added to the medium only (control). All compounds were diluted in 200 μl of serum-free DMEM, and the same solution with solvent diluents was used as control. After treatment, the cells were stimulated with TsV (50 μg ml^−1^) under the same experimental conditions and after 24 h at 37 °C in a humidified atmosphere 5% of CO_2_, the supernatants were collected for IL-1β quantification.

### Quantitative PCR with reverse transcription

RNA was extracted using a guanidine-based column method, according to the manufacturer protocol (Purelink, Ambion) and the quantity of RNA was determined by means of a fluorometric assay (Qbit, Invitrogen, Carlsbad, CA, USA). Complimentary DNA (cDNA) was synthesized from 1 μg of total RNA (High Quality cDNA Reverse Transcriptase Kits, Applied Biosystems, Foster City, CA, USA). Aliquots (2 μl) of the total cDNA were amplified by quantitative reverse transcriptase-polymerase chain reaction (quantitative PCR with reverse transcription (qRT–PCR)) using TaqMan primers for *Ptgs2*, *Ptges2*, *Ptger1*, *Ptger2*, *Ptger3*, *Ptger4*, *Alox5*, *Alox5ap*, *Ltb4r1* and *Ltb4r2* in a StepOne Plus machine (Applied Biosystems). *Gapdh* and *Actb* were used as reference genes. Reactions were performed in duplicate and amplification was performed under the following conditions: denaturation at 95 °C for 2 min; followed by 40 cycles of 95 °C for 2 s and 60 °C for 20 s. The results were normalized to the expression levels of the endogenous internal controls, *Actb* and *Gapdh*. The ΔΔCt method was used for the analysis, and the data were expressed as *n*-fold difference relative to the control.

### RNA interference using silencing RNA (siRNA)

J774.1 macrophages (1 × 10^5^) were seeded at 60–70% confluence and transfected with 60 pmol of *Ltb4r1* (sc-42587) and *Ltb4r2* (sc-45323) siRNAs (Santa Cruz Biotechnology, Santa Cruz, CA, USA) using Lipofectamine as the transfection reagent (Life Technologies, Carlsbad, CA, USA) and Opti-MEM reduced serum medium for culture (Life Technologies). Controls included non-targeting siRNA (scrambled and fluorescent) and no siRNA (with and without Lipofectamine). After 48 h of transfection, cells were pre-treated or not with LTB_4_ (100 nM; Cayman Chemicals) for 10 min and then stimulated with TsV (50 μg ml^−1^) for 6 h. Efficiency of transfection was 74% for *Ltb4r1* and 75% for *Ltb4r2*, as determined by qRT–PCR.

### Measurement of intracellular cAMP

For intracellular cAMP measurement, 1 × 10^6^ macrophages (peritoneal or J774.1) per well (48-well plate) were incubated with 1 ml of DMEM-serum free with or without PGE_2_ (1 μM) for 3 min, followed or not by TsV (50 μg ml^−1^) for 5 min at 37 °C in 5% CO_2_. In addition, cells were treated or not with indomethacin (10 μM), EP2 antagonist- AH6809 (1 μM) for 30 min before PGE_2_ (1 μM), or TsV (50 μg ml^−1^). In other sets of experiments, cells were pre-treated or not with LTB_4_ (10 nM) for 1 min, and then stimulated with PGE_2_ (1 μM) or TsV (50 μg ml^−1^). LTB_4_, PGE_2_, AH6809 from ethanol stock solution were diluted in the cell culture medium and the same concentration of ethanol (maximum 0.1%) was added to medium only (control). All stimuli (or ethanol) were diluted in serum-free DMEM medium. Culture supernatants were aspirated and the cells were lysed by incubation for 10 min with 0.1 M HCl at room temperature, followed by disruption using a cell scraper^27^. Intracellular cAMP was quantified by ELISA, using an acetylation protocol, according to the manufacturer (Enzo Life Sciences, Farmingdale, NY, USA).

### Immunoassay of phosphoproteins

To assess the levels of transcription factors (phosphoproteins) in J774.1 macrophages, a sandwich ELISA kit was used (PathScan Phospho-NFκB p65 (Ser536) and Total NFκB p65 ELISA Kit—Cell Signaling, Danvers, MA, USA). J774.1 macrophages were plated in 24-well micro-culture plates at a density of 2 × 10^6^ cells per well in serum-free DMEM medium and cultured at 37 °C in a humidified 5% CO_2_ atmosphere for 2 h. The cells were then stimulated with TsV (50 μg ml^−1^) for 2 h at 37 °C in a 5% CO_2_ atmosphere. In another set of experiments, PGE_2_ (10 μM) or forskolin (100 μM) were added in the culture 10 min before TsV stimulation. In addition, PKA-inhibitor H89 (25 μM) was added 2 h before TsV stimulation. After treatments, the adherent macrophages were washed with ice-cold PBS. Then, cell lysis buffer (Cell Signaling), containing protease and phosphatase inhibitors, was added to the cell culture. The lysates were centrifuged at 10,000*g* for 10 min at 4 °C in tubes and the supernatant was directly used or stored at −80 °C. The total protein content of the lysates was quantified, and normalized for used in ELISA kits according to the manufacturer's instructions (Cell Signaling). Briefly, the lysates were diluted with sample diluent and 100 μl of the lysate was added to wells that had been pre-coated with the primary antibody. The plate was incubated overnight at 4 °C and then washed. Next, the plate was incubated with the detection antibody and horseradish peroxidase-conjugated secondary antibody, with subsequent washes. The substrate 3,3′,5,5′-tetramethylbenzidine was added to the wells, the reaction was stopped with acid solution, and the absorbance of the samples was read at 450 nm. The results are expressed as relative percentages of the levels of phosphorylated NFκB to total NFκB, normalized by 100 mg of total protein.

### Dose–response experiments with TsV *in vivo*

In all experiments under all conditions, the mice were weighed before an i.p. injection of TsV. To determine the sublethal, lethal and excessive doses in WT mice (C57BL/6 and 129sv) or *Alox5*^*−/−*^, we tested TsV at 60, 120, 180 and 360 μg kg^−1^ in 200 μl of PBS and the survival was monitored for 24 h. Death of each animal was recorded immediately and used to calculate the rate of survival (in percentage points). On the basis of these results (Supplementary Fig. 1), the TsV dose of 120 μg kg^−1^ was considered sublethal and used to assess lung inflammation 4 h later; 180 μg kg^−1^ was designated as lethal, and 360 μg kg^−1^ as a superdose. These doses were used in survival experiments (with or without treatment) as indicated below. The animals were monitored for 8, 12 or 24 h. The animals that were inoculated with 200 μl of PBS (i.p.) served as negative controls. The mice were killed with an overdose of a solution of 20% ketamine and 10% xylazine.

### *In vivo* experiments and drug treatment

*IL-1r*^*−/−*^, *Casp1/11*^*−/−*^ and C57Bl/6 (WT) mice without treatment were inoculated with a sublethal or lethal dose of TsV (or PBS) as described above. *Alox5*^*−/−*^ mice and 129sv mice were pre-treated or not with IL-1 receptor antagonist (IL-1Ra) (ref. 63) at 10 mg kg^−1^, i.p., 1 h before and again 1 h after the sublethal or lethal TsV injection. IL-1Ra was kindly provided by Dr Stephen Poole, from the National Institute for Biological Standards and Control (South Mimms, Hertfordshire, UK). In a specific experiment, the mice were either treated or not treated with MK886 (5-LO inhibitor, 5 mg kg^−1^ i.p., in 200 μl of 1% alcohol in water)^64^, indomethacin (COX1/2 inhibitor, 2 mg kg^−1^ i.p. in 200 μl of Tris[hydroxymethyl]aminomethane-HCl; TRIS-HCl, pH 8.2)^65^, SC-560 (selective COX1 inhibitor, 3 mg kg^−1^ i.p., in 200 μl of PBS, Sigma-Aldrich)^66^, celecoxib (COX2 inhibitor, 5 mg kg^−1^ i.p., in 200 μl of water)^64^ or EP2 antagonist (AH6809, 5 mg kg^−1^ i.p., in 200 μl of PBS, Cayman Chemical)^67^. The drugs (MK886 or indomethacin) or vehicles were administered four times, at 4 h and 0.5 h before and again 4 and 8 h after the lethal dose of TsV. The others drugs (SC-560, celecoxib, and EP2 antagonist) or vehicles were administered 1 day and again 1 h before the i.p. injection of lethal dose of TsV (180 μg kg^−1^). In other experiments, the *Alox5*^*−/−*^ mice were treated or not with LTB_4_ (50 ng per mice, intranasal (i.n.) administration, in 20 μl of PBS, Cayman Chemical)^68^. The LTB_4_ or vehicle (PBS+0.05% of ethanol) were administered 2 h and 0.5 h before the dose lethal of TsV (180 μg kg^−1^). The lungs were excised immediately after death or from mice survivors that were killed 8–12 h after the injection of TsV or vehicle. In some sets of experiments, two groups of mice were inoculated with PBS or a sublethal (120 μg kg^−1^) dose of TsV and, in only one, BAL fluids were collected 4 h later, to count the total cell number and neutrophils, as described previously^47^. In the other group, without BAL, the lungs were excised and weighed and 2 mg of tissue was homogenized in 2 ml of incomplete RPMI. After centrifugation (400*g* for 10 min), the supernatants were transferred to new tubes, split into two samples of 1 ml and stored at −80 °C until use. One sample was used for IL-1β and protein quantification analysis and the other for PGE_2_ and LTB_4_ measurement. For analysis of MPO activity, one lobule of a lung was cut out, immediately frozen in liquid nitrogen, and stored at −80 °C until use. In the therapeutic protocol, the lethal dose (180 μg kg^−1^) and superdose (360 μg kg^−1^) of TsV were injected and indomethacin (2 mg kg^−1^ i.p.) or vehicle were administered either 15 or 30 min after and again 4 and 8 h later. Mice survivors were killed 12 h after the envenomation.

### Quantification of inflammatory markers

IL-1β present in the supernatant of a lung homogenate or cell culture and IL-6 in cell culture were quantified using IL-1β (BD Biosciences, Franklin Lakes, NJ, USA or R&D Systems) and IL-6 (R&D Systems) ELISA kits. Lipids were purified from 1 ml of filtered supernatants from the lung homogenates using Sep-Pak C_18_ cartridges (Waters Corp). Measurement of LTB_4_ and PGE_2_ levels was performed by means of an EIA assay (Enzo Life Sciences). The supernatants of the lung homogenates were used for the measurement of MPO activity as described previously^69^ and the quantification of total protein was conducted using the Coomassie Protein Assay Reagent (Pierce Chemical). In one set of experiments, peritoneal cavity fluid and 1 ml of peripheral blood were also collected to count total cells and neutrophils, using a Neubauer chamber and cytospin preparation. Immediately after an animal died or in mice survivors, the lungs were always weighed before processing and these data were used for the calculation of the lung/body weight index to evaluate oedema. When indicated in figures, the samples of lung parenchyma were stained with H&E for histological analysis and blind observers performed the analysis. In one set of experiments, three mice received 200 μl of a 1% Evan blue solution (Sigma-Aldrich) intravenously 60 min before euthanasia and the lungs were excised, weighed and photographed.

### Statistical analysis

For comparison of multiple groups, we performed one-way analysis of variance (ANOVA) followed by Bonferroni's post-test. The differences between any two groups were evaluated using two-tailed Student's *t*-test. All calculations were performed in the GraphPad Prism 5.0 software (GraphPad, San Diego, CA, USA). Differences in survival were analysed using the log-rank test. Differences with *P*<0.05 were considered statistically significant.

## Additional information

**How to cite this article:** Zoccal, K. F. *et al*. Opposing roles of LTB_4_ and PGE_2_ in regulating the inflammasome-dependent scorpion venom-induced mortality. *Nat. Commun.* 7:10760 doi: 10.1038/ncomms10760 (2016).

## Supplementary Material

Supplementary Information Supplementary Figures 1-6 and Supplementary Table 1-2
