# Supplementary material for: Opposing roles of LTB4 and PGE2 in regulating the inflammasome-dependent scorpion venom-induced mortality
Source: Nat Commun. 2016 Feb 23;7:10760. doi: 10.1038/ncomms10760 (PMC4766425; doi:10.1038/ncomms10760)
Supplement: Supplementary Information — Supplementary Figures 1-6 and Supplementary Table 1-2 [file ncomms10760-s1.pdf]

Supplementary Information

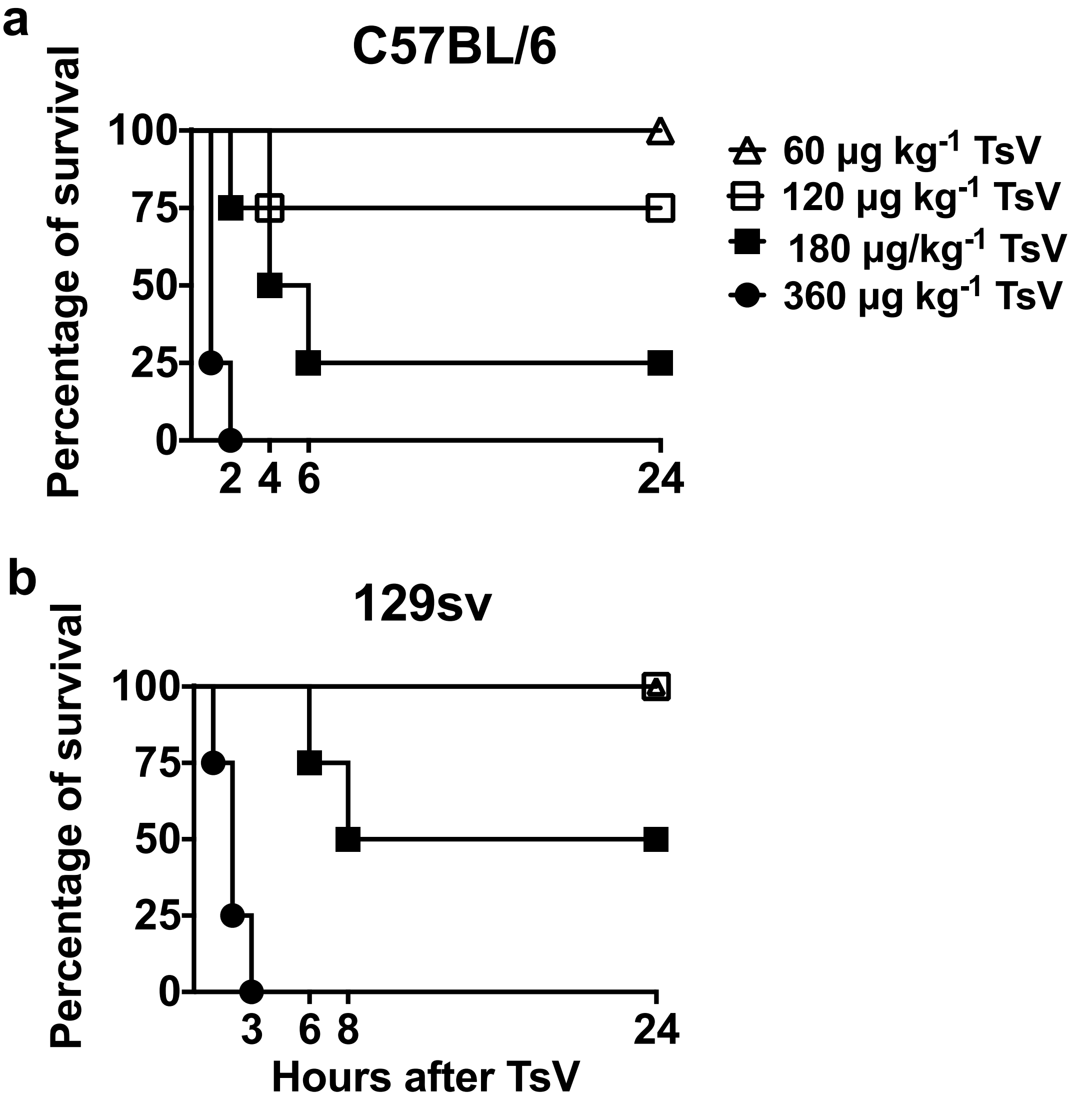

Supplementary Figure 1. Dose-response curves of *T. serrulatus* venom (TsV) in wild-type mice. (**a**) C57BL/6 and (**b**) 129sv mice were inoculated with TsV at 60, 120, 180, or 360 µg kg^{-1} and mortality was monitored for 24 h. The dose 120 µg kg^{-1} was considered sublethal, 180 µg kg^{-1} lethal, and 360 µg kg^{-1} a superdose. The dose-response experiment was performed once (n = 6).

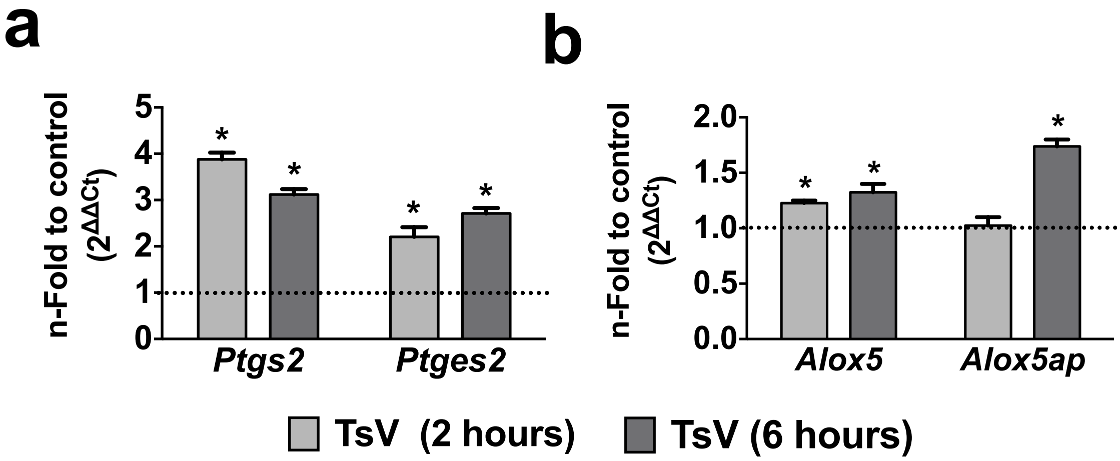

Supplementary Figure 2. TsV induces the expression of enzymes involved in the eicosanoid metabolism. Peritoneal macrophages from C57BL/6 mice were harvest to determine the gene expression of (**a**) *Ptgs2, Ptges2*, and (**b***) Alox5 and Alox5ap* by qRT-PCR after TsV-treatment (2 and 6 h) *in vitro*. The results were normalised to the expression levels of the endogenous internal control *Actb* and *Gapdh*. The ΔΔCt method was used for the analysis and expressed as n-fold to that of non-TsV stimulated macrophages. The results are presented as the fold-change measured from 2 independent experiments. Significant differences *(P* < 0.05) are marked with symbols and the error bars denote s.d. *Medium *versus* TsV, according to one-way analysis of variance (ANOVA) with Bonferroni's post-test.

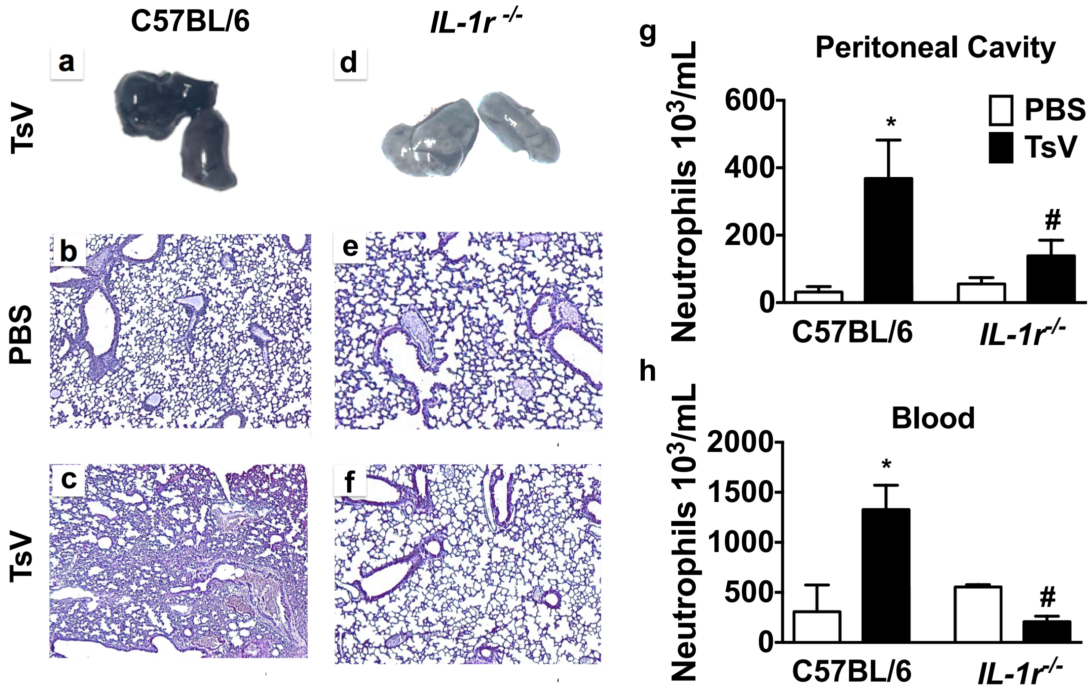

Supplementary Figure 3. *T. serrulatus* venom (TsV)-induced increase in the number of peritoneal and circulating neutrophils and lung inflammation are dependent on IL-1R signalling. (**a-f**) C57BL/6 (WT) mice and *IL-1r*$^{-/-}$ mice were inoculated i.p. with TsV (180 µg kg^{-1}) or PBS. (**b**,**c**,**e**,**f**) After 4 h, the mice were euthanised and the lungs were excised and prepared for staining with haematoxylin and eosin (H&E) for histological analysis (n = 3). Scale bars, 100 µm. (**a**,**d**) Additional three animals from each strain were inoculated intravenously with 200 µL Evans blue (1% solution) 60 min before euthanasia, for macroscopic visualisation of lung oedema. In other experiment, C57BL/6 (WT) mice and *IL-1r*$^{-/-}$ mice were inoculated i.p. with TsV (120 µg kg^{-1}) or PBS, and 4 h later, neutrophils in (**g**) a lavage fluid from the peritoneal cavity and in (**h**) blood samples were counted. Significant differences ($P < 0.05$) are marked with various symbols: *PBS *versus* TsV and #C57BL/6+TSV *versus* *IL-1r*$^{-/-}$+TSV, according to one-way analysis of variance (ANOVA) with Bonferroni's post-test (**g,h**). The data shown represent mean ± s.e.m. of one experiment on six mice.

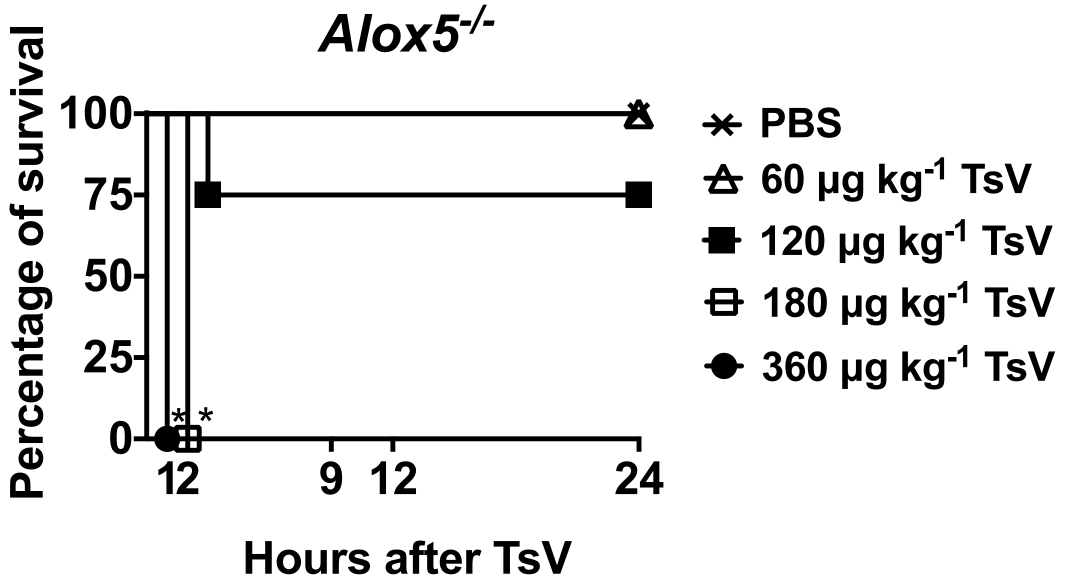

Supplementary Figure 4. Dose-response curves of *T. serrulatus* venom (TsV) in *Alox5-/-* mice. *Alox5*-/- mice were inoculated with TsV at 60, 120, 180, or 360 µg kg^{-1} or PBS and mortality was monitored for 24 h. The dose-response experiment was performed once ($n = 6$). $P < 0.05$, according to log-rank test. * PBS *versus* TsV.

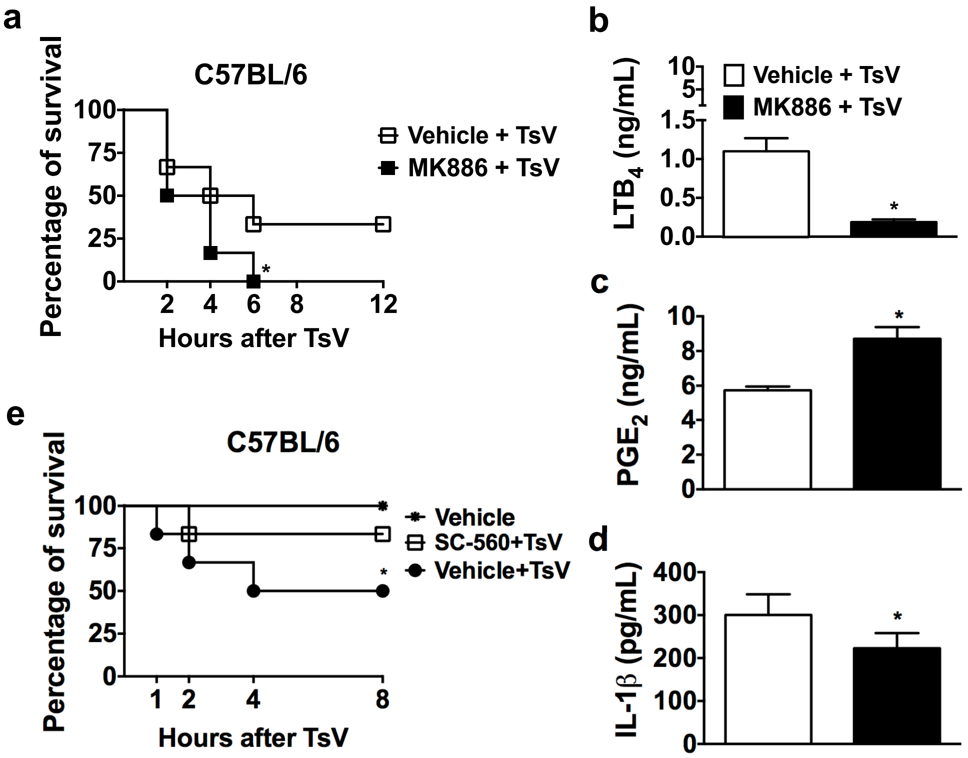

Supplementary Figure 5. Pharmacological inhibition of the 5-LO pathway increases, but COX1 inhibition partially decreases morality of wild-type mice inoculated with a lethal dose of *T. serrulatus* venom (TsV). (**a-d**) C57BL/6 wild-type mice were treated or not with MK886 (5 mg kg^{-1} i.p. in 200 μL of a 1% alcohol solution) or (**e**) SC-560 (3 mg kg^{-1} i.p. in 200 μL of PBS) or vehicle as described in *Methods* and were inoculated with a lethal dose of TsV (180 μg kg^{-1}). (**a**,**e**) Mortality was monitored for 8 or 12 h. Lungs were excised immediately after death or 12 h after the TsV injection for quantification of (**b**) LTB_4, (**c**) PGE_2, and (**d**) IL-1β by an enzyme-linked immunoassay (EIA) or ELISA. The asterisks mark significant differences ($P < 0.05$): (**a-d**) *Vehicle+TsV *versus* MK886+TsV or (**e**) Vehicle *versus* Vehicle+TsV. (**a**,**e**) Survival was analysed by the log-rank test and (**b**) differences between means ± s.d. of concentrations of the mediators were evaluated by means of Student's *t* test. The data shown correspond to one experiment with six mice.

a

Group	Weight of mice (g) (mean ± SEM)	Weight of lung (g) (mean ± SEM)	Lung weight/Mice (weight)
PBS (C57BL/6)	19.77 ± 0.389	0.123 ± 0.003	0.613 ± 0.015
TsV (C57BL/6)	19.84 ± 0.785	0.200 ± 0.004*	1.005 ± 0.048*
PBS (*IL-1r*$^{-/-}$)	20.59 ± 0.691	0.137 ± 0.005	0.700 ± 0.056
TsV (*IL-1r*$^{-/-}$)	20.94 ± 0.400	0.178 ± 0.004*#	0.864 ± 0.019*#

b

Group	Weight of mice (g) (mean ± SEM)	Weight of lung (g) (mean ± SEM)	Lung weight/Mice (weight)
PBS (C57BL/6)	19.03 ± 0.850	0.123 ± 0.002	0.652 ± 0.026
TsV (C57BL/6)	20.04 ± 0.511	0.263 ± 0.012*	1.351 ± 0.101*
PBS (*Casp1/11*$^{-/-}$)	19.20 ± 1.711	0.139 ± 0.004	0.666 ± 0.023
TsV (*Casp1/11*$^{-/-}$)	19.77 ± 0.55	0.110 ± 0.030#	0.575 ± 0.177#

c

Group	Weight of mice (g) (mean ± SEM)	Weight of lung (g) (mean ± SEM)	Lung weight/Mice (weight)
PBS (129sv mice)	20.60 ± 0.507	0.151 ± 0.011	0.734 ± 0.054
Vehicle + TsV (129sv mice)	20.27 ± 0.426	0.177 ± 0.012*	0.871 ± 0.052*
Indo + TsV (129sv mice)	20.18 ± 0.727	0.121 ± 0.002$	0.607 ± 0.031$
PBS (*5-lo*$^{-/-}$ mice)	21.08 ± 0.320	0.148 ± 0.012	0.698 ± 0.047
Vehicle + TsV (*5-lo*$^{-/-}$ mice)	20.42 ± 0.699	0.187 ± 0.020*	0.974 ± 0.045*
Indo + TsV (*5-lo*$^{-/-}$ mice)	19.88 ± 0.621	0.154 ± 0.007$	0.780 ± 0.042$

d

Group (C57BL/6 mice)	Weight of mice (g) (mean ± SEM)	Weight of lung (g) (mean ± SEM)	Lung Index/Mice (weight)
Vehicle + TsV	19.77 ± 0.390	0.173 ± 0.006	0.880 ± 0.036
MK886 + TsV	19.98 ± 0.640	0.124 ± 0.003$	0.627 ± 0.033$
Indo + TsV	20.33 ± 0.479	0.126 ± 0.003$	0.620 ± 0.014$

e

Group (C57BL/6 mice)	Weight of mice (g) (mean ± SEM)	Weight of lung (g) (mean ± SEM)	Lung Index/Mice (weight)
PBS	19.67 ± 0.338	0.137 ± 0.010	0.701 ± 0.063
Vehicle + TsV	20.90 ± 0.330	0.191 ± 0.006*	0.875 ± 0.057*
EP2 + TsV	19.82 ± 0.324	0.140 ± 0.004#	0.710 ± 0.022#
Celecoxib + TsV	21.26 ± 0.404	0.141 ± 0.005#	0.662 ± 0.020#

f

Group (C57BL/6 mice)	Weight of mice (g) (mean ± SEM)	Weight of lung (g) (mean ± SEM)	Lung weight/Mice (weight)
TsV (180 μg kg^{-1}) + Vehicle	20.30 ± 0.435	0.176 ± 0.005	0.870 ± 0.035
TsV (180 μg kg^{-1}) + Indo 15 min	20.37 ± 0.776	0.136 ± 0.002$	0.673 ± 0.029$
TsV (180 μg kg^{-1}) + Indo 30 min	19.80 ± 0.315	0.137 ± 0.005$	0.694 ± 0.034$
TsV (360 μg kg^{-1}) + Vehicle	20.62 ± 0.637	0.290 ± 0.004	1.415 ± 0.064
TsV (360 μg kg^{-1}) + Indo 15 min	20.08 ± 0.350	0.150 ± 0.004$	0.746 ± 0.026$
TsV (360 μg kg^{-1}) + Indo 30 min	20.62 ± 0.453	0.141 ± 0.004$	0.685 ± 0.016$

Supplementary Table 1. The lung/body weight index. The mice were weighed immediately before the injection of *T. serrulatus* venom (TsV). Immediately after death or at the end of the observation period, which was never more than 24 h, the entire lungs were weighed. For calculation of the index, lung weight was divided by body weight and the resulting number was multiplied by 100. Significant differences ($P < 0.05$) are marked with various symbols. The data are presented as mean ± s.d. *PBS *versus* TsV, #differences between strains, and $treated *versus* untreated mice, according to one-way analysis of variance (ANOVA) with Bonferroni's post-test (n = 6).

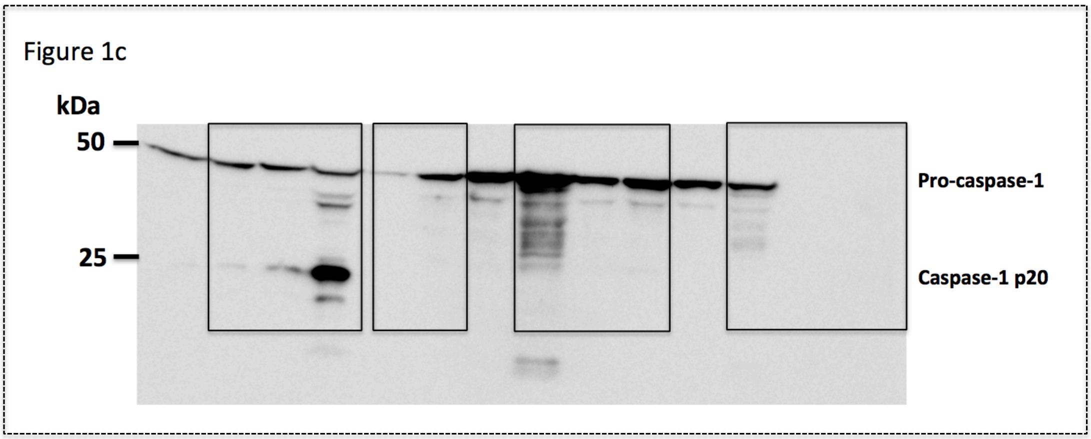

Supplementary Figure 6. Full immunoblot of main figure presented in this study.

Supplementary Table 2. List of the primers used in this study.

Gene	Sequence (5' to 3')
Ptgs2	Primer 1: GCGCAGTTTATGTTGTCTGTC<br>Primer 2: CAAGACAGATCATAAGCGAGGA<br>Probe: 6-FAM/TGATTTAAG/ZEN/TCCACTCCATGGCCCAG/3IABκFQ
Ptges2	Primer 1: GGCACTAATGATGACAGAGGAG<br>Primer 2: CCCGTGAGAAGGACTGAGAT<br>Probe: 6-FAM/AGGAAGGAG/ZEN/ACAGCTTGCAACAGC/3IABκFQ
Ptger1	Primer 1: ATCCGCTAGGCTCAGGTTA<br>Primer 2: AGTAGCTGGAGCAGGCA<br>Probe: 6-FAM/TGAGCAGCA/ZEN/CTGGCCCTCTTG/3IABκFQ
Ptger2	Primer 1: GAGGTTTCATCCATGTAGGCA<br>Primer 2: AGAGGAGAGAGGACTTCGATG<br>Probe: 6-FAM/ACCATCACC/ZEN/TTCGCCATATGCTCC/3IABκFQ
Ptger3	Primer 1: CCTTCTCCTTTCCCATCTGTG<br>Primer 2: GTGTGCTGTCCGTCTGTT<br>Probe: 6-FAM/TGCATTGCT/ZEN/CAACCGACATCTGATTGA/3IABκFQ
Ptger4	Primer 1: CTGATGTCTTTCACCACGTTTG<br>Primer 2: CATCTTACTCATCGCCACCTC<br>Probe: 6-FAM/TGCTCATCT/ZEN/GCTCCATTCCGCTC/3IABκFQ
Alox5	Primer 1: CCAGTCGTACTTTGAATCCGT<br>Primer 2: CCATCTGCCTGCTATATAAGAACC<br>Probe: 6-FAM/CCATTGCCA/ZEN/TCCAGCTCAACCAAA/3IABκFQ
Alox5ap	Primer 1: CTCCCAGATAGCCGAACAAAG<br>Primer 2: CAGAACTGCGTAGATGCGTA<br>Probe: 6-FAM/TGCCTCACA/ZEN/AACAGGTACATCAGTCC/3IABκFQ
Ltb4r1	Primer 1: CATGCCACCAGGAGAAGAAG<br>Primer 2: GGCACTAAGACAGATTCAAGGA<br>Probe: 6-FAM/CAGCCATCA/ZEN/AAAGGACAGGGTTTCC/3IABκFQ
Ltb4r2	Primer 1: GCTCAGTAGTGTCTCATTCCC<br>Primer 2: GGCTTCACCTGTCACCTT<br>Probe: 6-FAM/CCCCCAATG/ZEN/CTGTTCTCTCCC/3IABκFQ
Gapdh	Primer 1: GTGGAGTCATACTGGAACATGTAG<br>Primer 2: AATGGTGAAGGTCGGTGTG<br>Probe: HEX/TGCAAATGG/ZEN/CAGCCCTGGTG/3IABκFQ
Actb	Primer 1: GTACGACCAGAGGCATACAG<br>Primer 2: CTGAACCCTAAGGCCAACC<br>Probe: HEX/AGACCTTCA/ZEN/ACACCCCAGCCATG/3IABκFQ
